# Multifractal analysis of vegetation regulation on ecohydrological processes in a small watershed

**DOI:** 10.7717/peerj.20496

**Published:** 2026-01-12

**Authors:** Kai Shi, Bin Hu, Qiang Xiao, Songlin Tan

**Affiliations:** 1 College of Environmental Science and Engineering, China West Normal University, Nanchong, China; 2 Institute of Ecology, China West Normal University, Nanchong, China; 3 Nanchong Ecological and Environmental Monitoring Central Station of Sichuan Province, Nanchong, China

**Keywords:** Multifractal, Precipitation, Runoff, Small watershed

## Abstract

**Background:**

Runoff from small catchments facilitate water movement and a hydrologic balance across an area. In the watershed hydrological cycle, precipitation serves as the primary source of runoff, while runoff represents a delayed response to precipitation. Vegetation plays a crucial regulatory role in the relationship between precipitation and runoff through multiple ecohydrological mechanisms, including interception, infiltration regulation, and evapotranspiration. In different small watersheds, the dominant coupling mechanisms between precipitation and runoff exhibit clear temporal-scale dependence due to the variability of meteorological conditions and vegetation dynamics. Moreover, these interactions are strongly influenced by topographic features, vegetation cover, and soil composition, resulting in considerable uncertainty in the interrelationships among precipitation, runoff, and vegetation.

**Methods:**

We investigated the nonlinear relationship between precipitation and runoff at various time scales, drawing on long-term observational data (2017–2022) from the Quxi River catchment in China. We used the ensemble empirical mode decomposition (EEMD) and multifractal detrended cross-correlation analysis (MF-DCCA) to explore scale-dependent dynamics. The multifractal parameter was applied to reveal how water retention in the Quxi River small watershed varies across scales. To explore seasonal vegetation effects, we further conducted sliding window and Pearson correlation analyses.

**Results:**

EEMD, detrended fluctuation analysis (DFA), and MF-DCCA analyses were applied to runoff, precipitation, and vegetation cover data in the Quxi River watershed, China. EEMD revealed that high-frequency modes of precipitation and runoff, with a ∼2-week cycle, explained significant data variance. DFA showed precipitation as a random process, while runoff exhibited long-term persistence. MF-DCCA confirmed multifractal characteristics in precipitation-runoff coupling, with the multifractal parameter quantifying hydrological responses. Correlation coefficients between the multifractal parameter and fractional vegetation cover (FVC) were −0.07 (spring), 0.54 (summer), 0.34 (autumn), and 0.42 (winter), indicating vegetation’s moderating effect, especially in summer. Although both spring and summer have substantial precipitation exceeding 1,100 mm, the effects of vegetation dynamics on the watershed’s water retention capacity differ significantly between the two seasons. This is attributed to the vegetation type characteristics of the small watershed. This novel approach, integrating remote sensing and multifractal analysis, quantified vegetation’s regulation of watershed hydrology, offering a robust method to assess water retention capacity. It supports ecological restoration, forest management, and sustainable development in small watersheds, adaptable to regions with large hydraulic projects, enhancing ecosystem stability and biodiversity.

## Introduction

Small watershed runoff is closely related to the regional water cycle and plays an important role in maintaining regional ecological functions (Geng et al., 2010). In small watersheds, mountainous terrain blocks large-scale meteorological disturbances. Vegetation regulates the local redistribution of water and heat through interception, water storage, and transpiration, forming a unique small watershed climate, which significantly affects runoff formation. Therefore, an in-depth study of the relationship between precipitation and runoff in small watersheds is conducive to understanding the local climate adaptability of small watersheds, which will provide an important scientific basis for evaluating vegetation restoration and improvement of ecological service functions in small watersheds (Beniston, 2003).

Regional climate change will have different short-term and long-term impacts on small watershed runoff, mainly in terms of hydrological response intensity and processes. In areas with scarce vegetation, short-term heavy precipitation is difficult to intercept by plants, making such regions prone to flooding and causing a rapid hydrological response of runoff to rainfall (Machado, Oliveira & Lois-González, 2019). In contrast, a long-term increase in precipitation can alter the hydrothermal conditions of small watersheds, promoting vegetation growth and recovery. As plant root systems develop, their capacity to intercept, absorb, and store water is enhanced, which helps conserve water resources, reduce surface runoff, and facilitate deeper soil infiltration (Lyu et al., 2023). Meanwhile, vegetation transpiration also increases, further regulating runoff and mitigating the hydrological response of runoff to precipitation. These processes collectively strengthen the ecological service function of small watersheds.

The contrast between short- and long-term runoff responses highlights the complex interactions between rainfall, runoff, and vegetation in the local environment. Specifically, vegetation canopies intercept a portion of rainfall and return it to the atmosphere through evaporation, thereby directly reducing the amount of water contributing to runoff (Li et al., 2025). In addition, precipitation characteristics (such as type and intensity), vegetation transpiration, and root system structure all influence the interactions among rainfall, runoff, and vegetation, introducing a certain degree of uncertainty (Chang, Li & Lai, 2023; Dunne, Zhang & Aubry, 1991; Ni et al., 2022). Studies have found that in watersheds with low storage capacity, changes in vegetation cover can lead to a 30.7 ± 22.5% variation in annual runoff, potentially exceeding the influence of climate factors (Gan, Liu & Sun, 2021). It has been demonstrated that the non-stationarity of the precipitation–runoff relationship is primarily driven by endogenous mechanisms, such as groundwater levels, baseflow, and vegetation. The impacts of these endogenous mechanisms may change under climate change or drought events, resulting in seasonal-scale uncertainties in the interactions among precipitation, runoff, and vegetation (Deb, Kiem & Willgoose, 2019). However, due to the complexity of watershed characteristics, current research has not resolved the uncertainties inherent in these interactions.

Due to the temporal variability in meteorological conditions and vegetation, the main correlation mechanism between precipitation and runoff exhibits time-scale dependence (Zhang, Viglione & Blöschl, 2022b). The hydrological response of precipitation transforming into runoff can occur at time scales ranging from seconds to hours. Heavy rainfall often did not penetrate the soil or become absorbed by plants quickly enough, causing runoff to rise dramatically within a few hours (Trenberth et al., 2003). Hydrologic responses can also manifest themselves at longer time scales, such as months, years, or even decades. El Niño-Southern Oscillation (ENSO) is a large-scale atmospheric circulation pattern that occurs every three to seven years. It has a strong influence on precipitation patterns, and precipitation patterns in turn affect runoff (Trenberth, 2019). During the ENSO process, the variation in precipitation usually increases for low and average amounts and decreases for high and variable amounts (You et al., 2021). Moreover, the relationship between precipitation and runoff is influenced by the choice of the study period. La Torre Torres et al. (2011) found that in the low-lying basins of the southern United States, the precipitation–runoff relationship displayed pronounced seasonal variation, shaped by local topography, soil, and climate conditions. Specifically, the runoff-to-precipitation ratios were lower during dry seasons (summer and autumn) and higher during wet seasons (winter and spring). These findings suggest that the correlation between precipitation and runoff evolves with temporal scale and may also differ across spatial scales. As a result, conventional correlation-based methods are not able to capture the intrinsic link between precipitation and runoff (Jiang, Xu & Xiong, 2022). The differences in the mechanisms underlying precipitation and runoff formation lead to complex, multi-scale, and nonlinear variations between the two (Lima & Lall, 2010).

Multifractals, as an important theory and method in nonlinear science, can quantitatively characterize the variation patterns of variables across different scales, reflecting the multi-scale heterogeneity of hydrological response processes. The multifractal detrended cross-correlation analysis (MF-DCCA) is a key technique within multifractal analysis. It is a nonlinear time series method that quantitatively describes the cross-scale correlation structure between different variables (Zhou, 2008). Studies have shown that precipitation usually does not show a long-term correlation, but runoff does, reflecting the spatial and temporal differences between the two (Kantelhardt et al., 2006). Precipitation is the primary source in the hydrologic cycle of a watershed, while runoff usually occurs after a delay in precipitation. This process typically exhibits nonlinear characteristics (Li et al., 2024a). Since the MF-DCCA method effectively handles spatio-temporal heterogeneity and nonlinear features, it is hypothesized that the method can reveal long-term correlated effects of precipitation and runoff over multiple time scales. These long-term interactions have the characteristics of scale invariance and self-similarity, which are the hallmarks of fractal behavior. This indicates that the strength of the interactions between precipitation and runoff exhibits scale invariance across time, meaning that similar dynamic statistical distribution patterns are observed at different temporal scales. On a certain time scale, the strong long-term correlation indicates that at a specific moment, the interaction between precipitation and runoff may have a lasting impact on future hydrological responses (Bae, Jung & Chang, 2008).

To address the mode mixing problem in the Empirical Mode Decomposition (EMD), where a single intrinsic mode function contains signal components with significantly different scales, or components of the same scale are spread across different intrinsic mode functions (IMFs), Wu & Huang (2009) proposed the ensemble empirical mode decomposition (EEMD). This method, based on the signal’s adaptive nature, decomposes the original sequence into a finite number of signal components, known as Intrinsic Mode Functions. A key innovation of EEMD is the intentional addition of Gaussian white noise, which helps reduce mode mixing, thereby improving the accuracy and robustness of the EEMD decomposition. EEMD has been widely recognized as a powerful tool for analyzing nonlinear and non-stationary time series.

Therefore, this study combines multiple methods, including EEMD, detrended fluctuation analysis (DFA), and MF-DCCA, aiming to use nonlinear analysis to explore the nonlinear relationships of hydrological responses across multiple time scales. These nonlinear methods, based on long-term hydrological observations and remote sensing time series, can be directly applied to the original monitoring data without the need to predefine model parameters. Consequently, they naturally avoid the uncertainties arising from the complex interactions among multiple drivers, such as precipitation, runoff, and vegetation. When the time series data are sufficiently long, nonlinear methods can effectively investigate the intrinsic nonlinear associations of hydrological responses across multiple time scales (Liu et al., 2018). To date, no studies have applied this approach to investigate water retention capacity in small watersheds.

The Quxi River Basin is located in the tailwater area of the Three Gorges Reservoir, the world’s largest hydropower station. Influenced by the complex mountainous terrain, the river follows a meandering course and exhibits distinctive geomorphological features. This area is influenced by a monsoon climate, in which interactions among local precipitation, runoff, and vegetation form a distinctive hydrological response mechanism. The Quxi River Basin was analyzed in this case study, combining remote sensing precipitation imagery, ground-measured runoff data, and vegetation coverage data to investigate the multiscale coupling characteristics of precipitation, runoff, and vegetation in a small watershed under local climatic conditions using EEMD and MF-DCCA methods. By integrating the multifractal approach with vegetation dynamics, it is possible to achieve dynamic monitoring and diagnosis of ecological risks in the Quxi River Basin within the Three Gorges Reservoir area, reveal the mechanisms by which vegetation dynamics influence hydrological responses in small watersheds, and provide a scientific basis for the quantitative assessment of the ecosystem service function of water retention in small watersheds.

## Materials & Methods

### Study area

**Figure 1:**
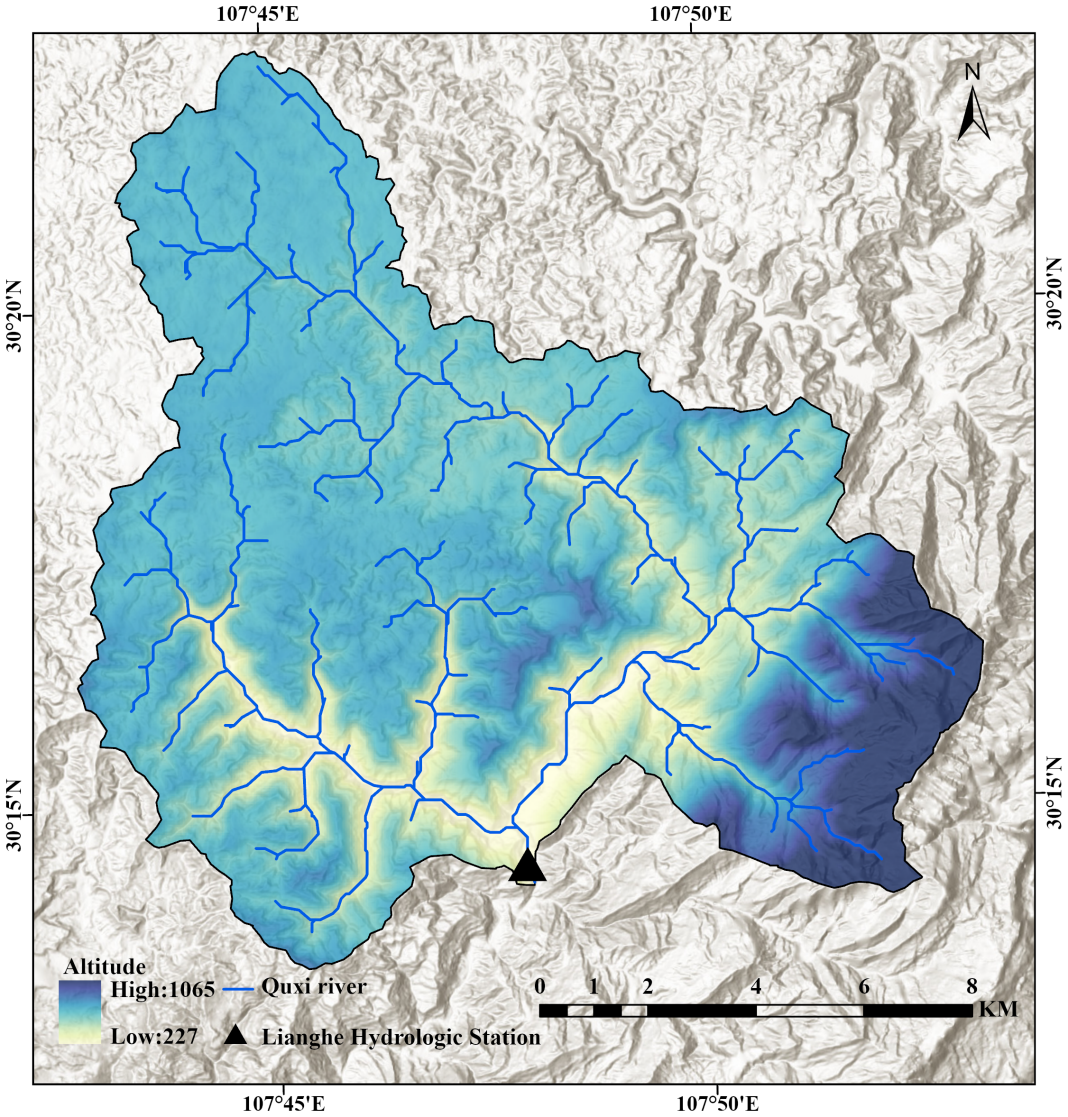
Geographical location of the Quxi River Basin in Zhongxian County, Chongqing, China.

The Quxi River is a primary tributary on the left bank of the Yangtze River (Fig. 1). The main stream originates in Daoguan Township in Zhongxian County, Chongqing, China, and flows through the Lianghe Hydrological Station (30°14′N, 107°47E), with a controlled watershed area of 62.6 km^2^. The Quxi River Basin (30°13′–30°23′N, 107°43′–107°53′E) is located in the southwestern part of Zhongxian County, Chongqing, China, and is primarily characterized by hilly terrain. The overall topography slopes from north to south, with the region surrounded by Jinhua, Fangdou, and Maowei Mountains, forming a relatively enclosed geomorphological unit.

 The Quxi River small watershed is located in the Three Gorges Reservoir area. Research on this region contributes to a systematic and quantitative assessment of the impacts of large-scale hydraulic engineering projects on small watersheds.

The region experiences a subtropical monsoon climate, with hot, rainy summers and cold, dry winters. However, high-altitude areas are occasionally affected by cold waves in winter, which may lead to snowfall. The annual average precipitation is 1,072 mm, with significant interannual variability and an uneven seasonal distribution, primarily concentrated in summer and autumn.

The Quxi River Basin features a relatively enclosed topography, with large-scale water vapor transport hindered by the unique mountainous terrain, resulting in minimal direct influence from external climatic conditions. The watershed itself exhibits high vegetation cover, with cultivated crops being the dominant vegetation type. This enclosed topographic feature and high vegetation coverage endow the small watershed with distinct local climatic characteristics.

The industrial, agricultural, and human activities in the Quxi River Basin are shaped by its unique geographical and climatic conditions. The basin has a humid climate, providing favorable conditions for agricultural development, particularly supporting the rapid expansion of the citrus industry. The Zhongxian County 2024 Statistical Yearbook shows that the cumulative transaction value of citrus has exceeded 10 billion yuan by the end of 2024.

### Data sources

The daily runoff data used in this study were automatically recorded by the Lianghe Hydrological Station (30°14′N, 107°47E) in the Quxi River Basin, Zhongxian, Chongqing, China, covering the period from 2017 to 2022.

In this study, we used the CHM_PRE daily precipitation dataset provided by the National Qinghai-Tibet Plateau Science and Technology Data Center (TPDC). The dataset spans the years 2017 to 2022 and has a spatial resolution of 0.1°. CHM_PRE is derived from observational data collected from 2,839 meteorological stations within and around China, covering a long-term period from 1961 to 2022 (Gou et al., 2021; Han et al., 2023; Miao et al., 2022). Due to its high quality and extensive coverage, this dataset has been widely adopted in hydrological and climate change studies (Tang, Wang & Chen, 2024; Wei et al., 2025). ArcGIS was used to clip the precipitation data at the boundary of the Quxi River Basin, allowing for the extraction of daily raster data within the study area. Precipitation values from three grid points inside the basin were then aggregated to calculate total daily precipitation for the years 2017 to 2022, forming the basis for the daily precipitation time series.

We retrieved NDVI data from NASA’s MOD13A3 product, which offers global monthly values at a 1-kilometer resolution. Using a Python-based pixel calculation method, we estimated the Fractional Vegetation Cover (FVC) for each month from 2017 to 2022. The FVC results were then spatially clipped to the Quxi River Basin using ArcGIS, allowing us to extract the monthly vegetation coverage for the study area and build a corresponding time series composed of monthly FVC values.

The DEM data used in this study is the ASTER GDEM (Advanced Spaceborne Thermal Emission and Reflection Radiometer Global Digital Elevation Model), with a spatial resolution of 30 m. The slope was calculated using ArcGIS. The vegetation type group data used in this study were obtained from the National Glacier, Permafrost, and Desert Science Data Center (https://www.ncdc.ac.cn), with a spatial resolution of 10 km. The data were resampled to a 30 m resolution using ArcGIS to analyze the distribution of different vegetation types in the Quxi River Basin. Soil type data used in this study were obtained from the Resource and Environmental Science Data Center (http://www.resdc.cn), with a spatial resolution of one km. The data were resampled to a 30 m resolution using ArcGIS to analyze the distribution of different soil types in the Quxi River Basin.

### Methods

#### Ensemble empirical mode decomposition

Ensemble empirical mode decomposition (EEMD) is a decomposition method proposed by Wu & Huang (2009) for processing nonlinear and non-stationary signals. The core idea is to add Gaussian white noise, which follows a normal distribution and has the same length as the original time series, making the signal changes exhibit continuity across time scales. This decomposition method yields multiple intrinsic mode functions, each representing a characteristic oscillatory mode in the data (Wu & Huang, 2009). Its adaptability has led to a wide range of applications in ocean dynamics, biomedical signal analysis, and climate variability studies (Gao et al., 2023; Guo et al., 2025; Han, Lin & Xu, 2017). In this study, the EEMD method was applied separately to the precipitation and runoff time series. Gaussian white noise of the same length as the original sequence was added, followed by multiple EEMD decompositions, and the resulting intrinsic mode functions (IMFs) were averaged to reduce the influence of noise. The standard deviation of the white noise was set to 0.2, and the number of ensembles *M* was set to 100, as suggested by Wu & Huang (2009). The final decomposed results were used to quantitatively analyze the variation characteristics of precipitation and runoff across multiple time scales. The specific algorithm can be referred to in the literature (Du et al., 2021).

(1) \begin{eqnarray*}Y \left( t \right) =\sum _{j=1}^{m}{H}_{j} \left( t \right) +{r}_{m} \left( t \right) .\end{eqnarray*}

In Eq. (1), *Y*(*t*) is the original time series, *t* represents the time scale, *m* is the number of IMFs obtained from the decomposition, *r*_*m*_(*t*) is the residual term, and *j* denotes the frequency component. The IMF components *H*_1_(*t*), *H*_2_(*t*),…, *H*_*j*_(*t*) represent the fluctuation components at different time scales. These fluctuation components, namely IMF_1_, IMF_2_, and so on, correspond to oscillations with their periods gradually increasing from short to long.

#### Detrended fluctuation analysis

DFA is a nonlinear time series analysis method particularly well-suited for examining long-range correlations in non-stationary time series. In this study, the DFA method was applied separately to the precipitation and runoff time series. Through cumulative deviation processing, segment-wise least-squares detrending, and calculation and averaging of the variances of each subinterval, the standard DFA fluctuation function *F*(*n*) was obtained, quantitatively identifying the long-term persistence characteristics of precipitation and runoff across multiple time scales. The specific procedure can be referred to in the literature (Shi et al., 2018).

(2) \begin{eqnarray*}F(n)\propto {n}^{\mathrm{\alpha }}.\end{eqnarray*}

In Eq. (2), *F*(*n*) represents the detrended fluctuation function, *n* is the time scale, and α is the DFA exponent, which serves as the self-similarity parameter. If the *F*(*n*) data points exhibit a linear relationship with *n* on a log–log plot, there exists a power-law relationship *F*(*n*) ∝ *n*^α^. The slope of the fitted line obtained using the least squares method is the parameter α. The parameter α reflects whether the non-stationary time series has long-term persistence. If 0.5 < α < 1, the time series has long-term persistence; if α = 0.5, the time series does not have long-term persistence; if 0 < α < 0.5, the time series exhibits the opposite.

#### Multifractal detrended cross-correlation analysis

Multifractal detrended cross-correlation analysis is a method used to analyze the long-term correlation and multifractal characteristics between two non-stationary time series. The MF-DCCA method was applied, with precipitation as the independent variable and runoff as the dependent variable, involving cumulative deviation processing, segment-wise detrending, and calculation of the *q*-th order fluctuation functions, to quantitatively reveal the multiscale response of runoff to precipitation. The procedure can be referred to in the literature (Wu et al., 2021).

Finally, if there exists a long-term power-law cross-correlation between precipitation and runoff time series, then:

(3) \begin{eqnarray*}{F}_{q}(n)\propto {n}^{h(q)}.\end{eqnarray*}

In Eq. (3), *F*_*q*_(*n*) is the *q*-order fluctuation function, *n* represents the time scale, *q* denotes the order, and *h*(*q*) is the generalized Hurst exponent.

In this study, the value of *q* ranged from −20 ≤ *q* ≤ 20. This range allows for a detailed distinction between the scaling behaviors of large and small fluctuations, without causing divergence issues (Zhang et al., 2022a). When *q* =  − 20, the analysis is sensitive to extremely small fluctuations; when *q* = 20, it is sensitive to extremely large fluctuations. When *q* = 2, MF-DCCA is equivalent to DCCA (Shi et al., 2025), and *h*(*q*) represents the cross-correlation index in the DCCA method. *h*(2) = 0.5 indicates no correlation between the series; *h*(2) > 0.5 indicates a long-term power-law cross-correlation between precipitation and runoff, suggesting that an increase in precipitation may lead to an increase in runoff over a certain future time scale, meaning that the correlation between precipitation and runoff exhibits long-term persistence. On the other hand, *h*(2) < 0.5 implies the opposite.

If *h*(*q*) decreases monotonically with increasing *q*, then the cross-correlation between the precipitation and runoff series exhibits typical multifractal characteristics. The strength of multifractality can be quantified by the range of *h*(*q*):

(4) \begin{eqnarray*}\Delta h=\max \nolimits ~h \left( q \right) -\min \nolimits ~h \left( q \right) .\end{eqnarray*}

In Eq. (4), the larger the Δ*h*, the stronger the multifractality.

### Remote sensing inversion of vegetation coverage

Remote sensing-based inversion of vegetation coverage is a method that utilizes remote sensing technology to estimate and monitor vegetation coverage on the Earth’s surface. Based on the pixel dichotomy model, the Normalized Difference Vegetation Index $ \left( NDVI \right) $ can be converted into Fractional Vegetation Cover (*FVC*) as follows:

(5) \begin{eqnarray*}FVC= \frac{NDVI-NDV{I}_{soil}}{NDV{I}_{veg}-NDV{I}_{soil}} .\end{eqnarray*}

In Eq. (5), *FVC* represents the fractional vegetation cover (%), *NDVI*_*veg*_ is the *NDVI* value of areas fully covered by vegetation, and *NDVI*_*soil*_ is the *NDVI* value of areas completely lacking vegetation coverage.

## Results

### EEMD decomposition of precipitation and runoff time series

**Figure 2:**
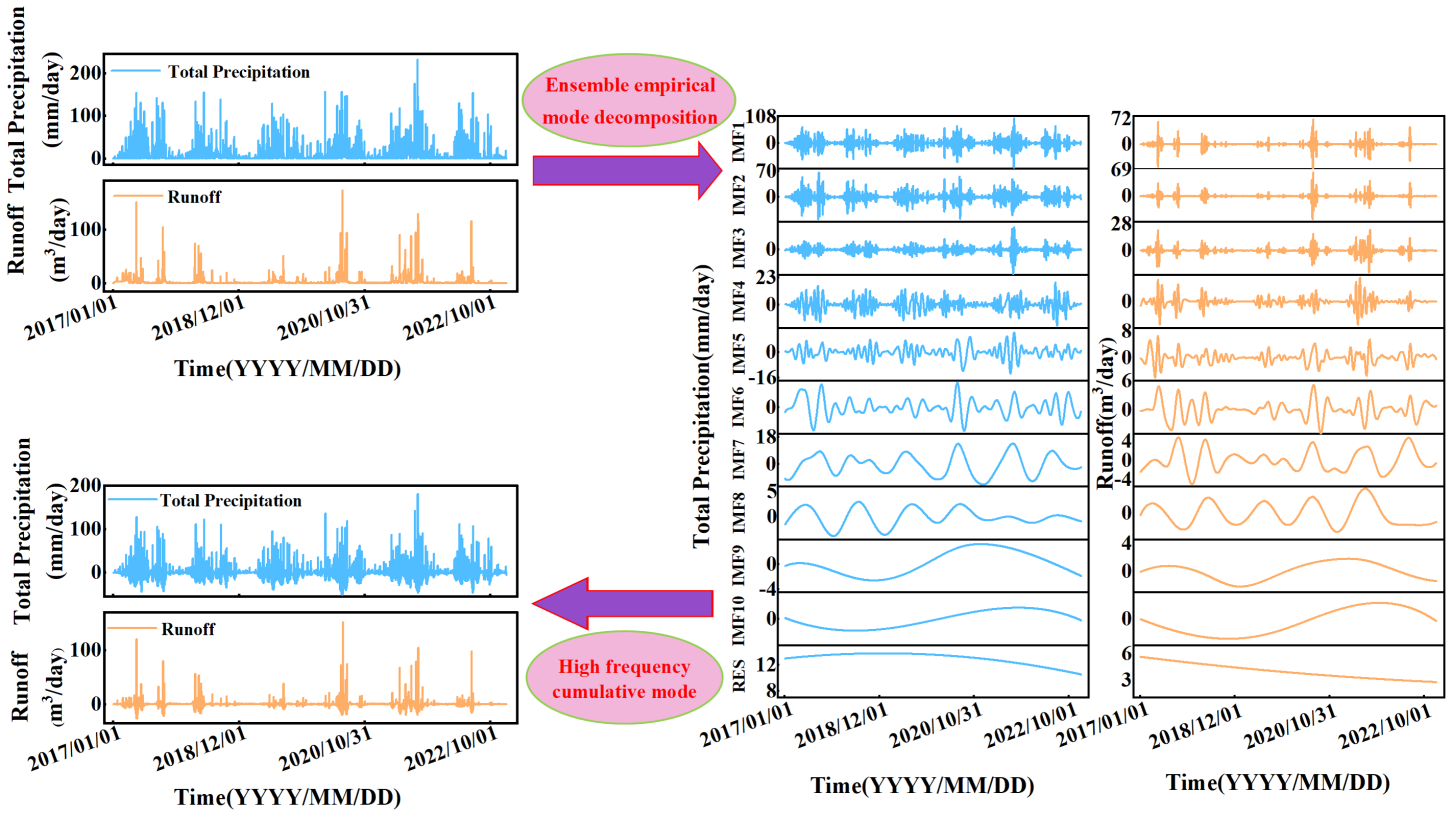
The EEMD decomposition results of precipitation and runoff series and the extraction results of the top three high-frequency sequences.

To better understand the multiscale evolution of precipitation and runoff time series, EEMD decomposition was applied to both original series from the Quxi River Basin for the years 2017–2022. This process yielded ten intrinsic mode functions (IMF1–IMF10) and one long-term trend component (RES). The decomposition results are shown in Fig. 2. These IMF components represent the fluctuation characteristics of the precipitation and runoff time series across different time scales, ranging from high to low frequency. The long-term trend component captures the overall variation in precipitation and runoff in the Quxi River Basin over the six-year period. The gradual downward trend observed in both precipitation and runoff suggests a potential correlation between the two variables.

Table 1 presents the average period contribution rate and variance contribution rate of the IMF components obtained from the EEMD decomposition of precipitation and runoff. These metrics reflect the temporal characteristics of each IMF component as well as the influence of their frequencies and amplitudes on the original time series. The precipitation and runoff time series contain typical periodicities such as daily, weekly, and monthly cycles, each with practical physical significance.

**Table 1:** The main statistical values of the precipitation and runoff IMF components in the Quxi River Basin, Chongqing, China, from 2017 to 2022.

IMF components	**Precipitation**		**Runoff**	
	Mean period (day)	Variance ratio (%)	Mean period (day)	Variance ratio (%)
IMF1	3.57	46.75	4.72	43.33
IMF2	8.21	24.73	10.53	29.53
IMF3	13.41	10.25	18.26	9.29
IMF4	31.75	4.58	29.21	5.69
IMF5	50.95	2.24	81.15	2.23
IMF6	109.55	2.01	109.55	2.46
IMF7	365.17	8.21	365.17	3.43
IMF8	365.17	0.48	365.17	2.21
IMF9	2,191	0.61	2,191	1.11
IMF10	2,191	0.02	2,191	0.18
RES	2,191	0.134	2,191	0.54

The oscillation intensities of different modes exhibit uneven temporal variations. As shown in Table 1, the first three components of precipitation and runoff account for a large proportion, with a cumulative variance contribution to the total variance approaching 80%. This indicates that short-period fluctuations have a significant impact, suggesting that short-term hydrological processes play a critical role in the precipitation and runoff dynamics in the Quxi River Basin.

Additionally, significance testing can be employed to determine whether each IMF component has physical meaning. The significance level is set to α = 0.01. If an IMF component lies on the critical line for α = 0.01, it is considered to have passed the 99% significance test, indicating that the information it contains is statistically meaningful within the confidence level. Otherwise, the component is likely dominated by white noise.

In this study, after applying EEMD decomposition on the precipitation and runoff sequences in the Quxi River Basin and conducting significance testing, the first eight IMF components fell above the critical line for α = 0.01, thereby passing the significance test. This suggests that the first eight components derived from the decomposition of the precipitation and runoff sequences in the Quxi River Basin have physical meaning within the original sequences with 99% confidence. On the other hand, IMF9 and IMF10 did not pass the test, suggesting they contain considerable white noise. However, these components have long periods and low contribution rates, so their influence on the results is negligible.

### The DFA analysis of the precipitation and runoff series

**Figure 3:**
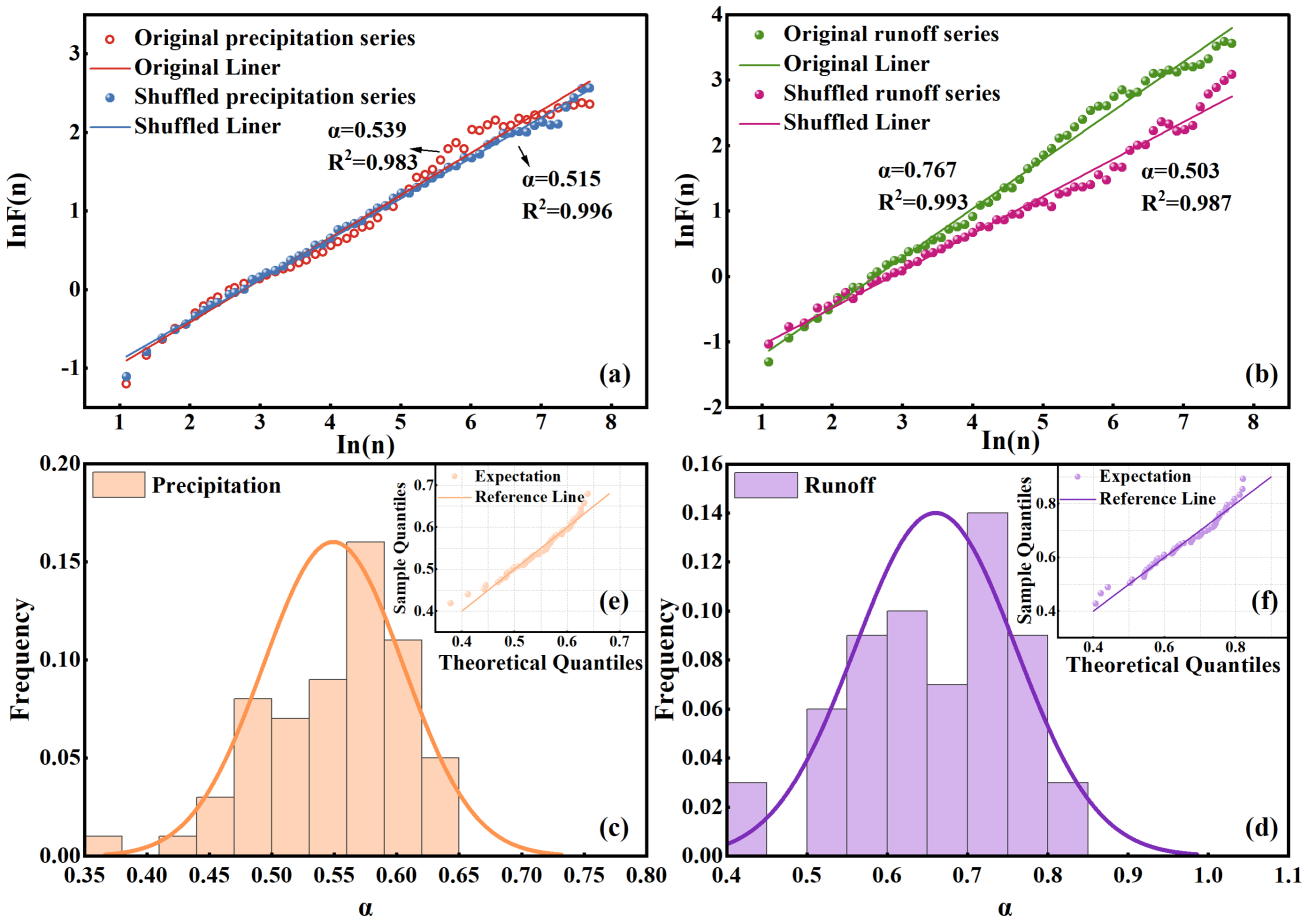
DFA analysis of precipitation and runoff series. Plots of (A) and (B) represent the lnF(n) ∝ ln(n) for the original and randomized sequences of precipitation and runoff data in the Quxi River Basin from 2017 to 2022; (C) and (D) show the frequency histograms of values for precipitation and runoff data overlaid with normal distribution curves; and (E) and (F) display the Q–Q plots of precipitation and runoff data.

To evaluate whether the non-stationary time series of precipitation and runoff in the Quxi River Basin exhibited long-term persistence, we used the DFA method. The analysis was conducted on the precipitation and runoff for the years 2017 to 2022. As shown in Figs. 3A and 3B, the relationship between ln*F*(*n*) and *lnn* for both precipitation and runoff displays strong linearity, indicating the presence of a power relationship. The slope of the fitted line, obtained using the least squares method, represents the DFA scaling exponent α. The α value for precipitation is 0.54, while that for runoff is 0.76. The DFA scaling exponent α reflects the temporal correlation structure of the series across different recurrence intervals. The α value of precipitation is close to 0.5, suggesting that precipitation in the Quxi River Basin lacks long-term persistence and behaves as an uncorrelated random process. In contrast, the α value for runoff is significantly greater than 0.5, indicating pronounced long-term persistence.

To verify whether the DFA method accurately reflects the long-term persistence characteristics of a non-stationary series, the previously used data were randomly shuffled, and DFA was reapplied to the reshuffled sequences. As shown in Figs. 3A and 3B, the α values of the randomized precipitation and runoff series are both close to 0.5, indicating that they behave as completely random processes with no long-term persistence. This confirms that the observed long-term persistence in the original runoff series is not an artifact of data distribution or trend errors, but is a genuine intrinsic property of the hydrological process.

To further investigate the dynamic evolution of long-term persistence in precipitation and runoff, we accounted for their significant temporal heterogeneity across different time scales, wherein the DFA scaling exponent α may vary over time. Therefore, a sliding window approach was employed. Using the precipitation and runoff data from January 1 to December 31, 2017, as the initial window, the DFA method was applied to calculate the α value. The window length was fixed at one year, and the window was then shifted forward by one month to compute the next α value. In this manner, a series of α values was obtained. By segmenting the entire time series from 2017 to 2022, a series of 61 α values was obtained. Figures 3C and 3D present histograms of the α values for precipitation and runoff, respectively, overlaid with normal distribution curves. The results show that the α values of precipitation were close to 0.5 overall, suggesting a lack of long-term persistence and indicating that precipitation behaves as a purely random process. In contrast, most α values of runoff exceeded 0.5, revealing a consistent characteristic of long-term persistence in runoff.

To further evaluate whether the α values follow a normal distribution, we used quantile–quantile (Q–Q) plots for verification, as shown in Figs. 3E and 3F. In a Q–Q plot, data points approximately aligning along a straight line indicate that the sample data may originate from a normal distribution, and it may be assumed that the data are normally distributed. As observed in the figures, the α values for both precipitation and runoff exhibited normal distribution. In this study, the mean α value for precipitation was 0.55, or close to 0.5, indicating that the precipitation series lacked long-term persistence and behaved as a near-random process. In contrast, the average α value for the runoff was 0.66, significantly greater than 0.5, suggesting that the runoff series possessed strong long-term persistence.

Figure 3D shows that the long-term persistence of runoff varied significantly across time scales. This suggests that runoff dynamics were subject to considerable uncertainty.

### Multifractal characterization of precipitation and runoff

To characterize the spatial–temporal variability characteristics and the nonlinear relationship between precipitation and runoff, we used the MF-DCCA method and analyzed the relationships based on the precipitation and runoff from 2017 to 2022.

**Figure 4:**
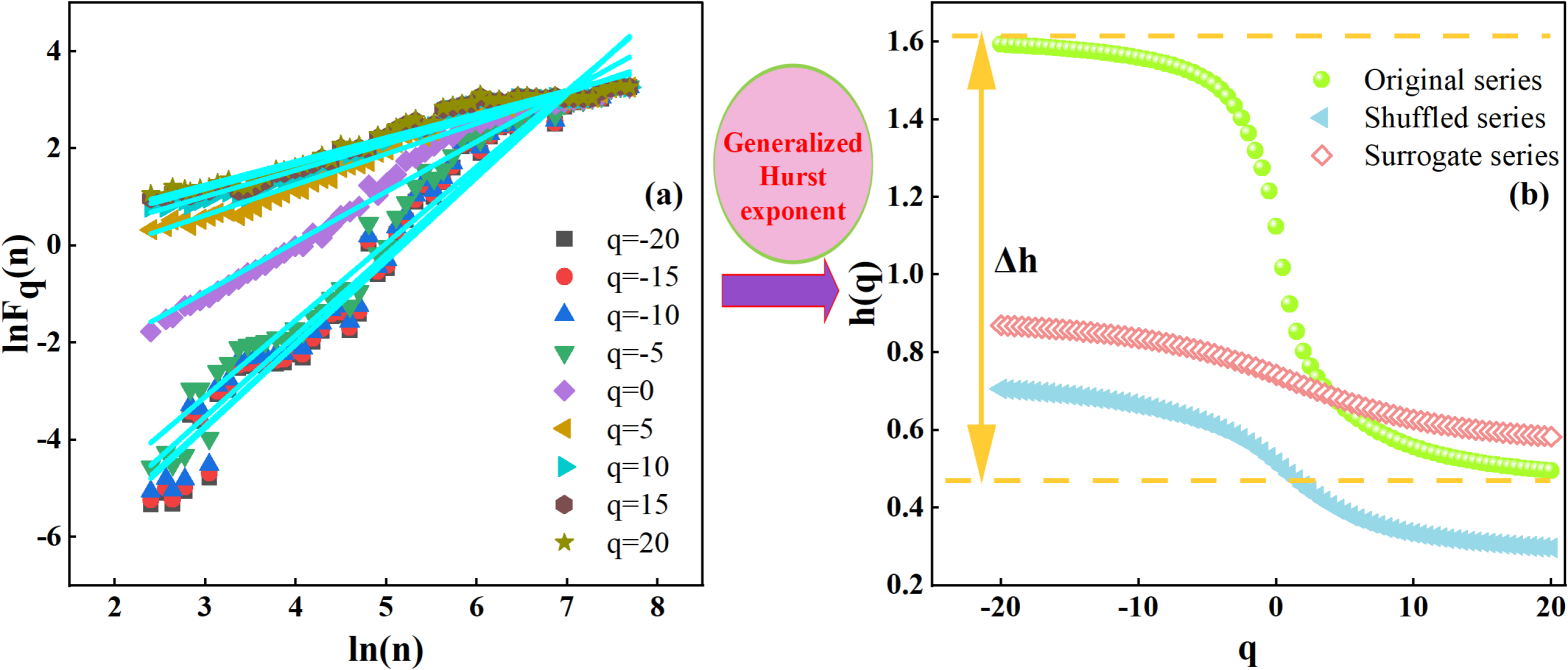
MF-DCCA analysis of precipitation and runoff series. (A) ln(n) lnFq(n)plot for precipitation and runoff series. (B) q-h(q) plot for original series, shuffled series, and random series. The parameter q is an order parameter that controls the sensitivity to fluctuations of different magnitudes.

Figure 4 displays a clear linear pattern on the log–log plot between the scale *ln*(*n*) and the detrended fluctuation function *lnF*_*q*_(*n*) for different q-values. This indicates a strong multifractal cross-correlation between precipitation and runoff in the Quxi River Basin. In particular, the plots of *ln*(*n*) against *lnF*_*q*_(*n*) at *q* = 0,  ± 5,  ± 10,  and ±20 all show similar straight lines that closely overlap. This indicates that the cross-correlation between precipitation and runoff is a power function. Furthermore, it suggests that over certain time scales, the cross-correlation between precipitation and runoff does not conform to a classical Markovian process, but instead decays gradually in a power-law manner over time. Therefore, the cross-correlation between precipitation and runoff exhibits multifractal scale invariance, reflecting a consistent relationship across temporal scales.

As shown by the green line in Fig. 4, when *q* = 2, the original series for the Quxi River Basin yields a generalized Hurst exponent of *h* = 0.80. This indicates a positive correlation between precipitation and runoff, which persists over time in the form of a power-law relationship over the time scale of the study period. This suggests that an increase in precipitation may lead to an increase in runoff within a certain future time scale, demonstrating the presence of long-term persistence in their cross-correlation. The multifractal strength (Δ*h*) of precipitation and runoff in the Quxi River Basin was subsequently calculated as 1.10, indicating a strong multifractal intensity in the cross-correlation between precipitation and runoff.

To identify the sources of multifractal behavior in the interaction between precipitation and runoff, phase randomization and random shuffling were applied separately to the original data. Then, the MF-DCCA method was used to analyze the surrogate and randomized series, obtaining the $q-h \left( q \right) $ function relationships for the original, surrogate, and randomized sequences. As shown in Fig. 4, the gap between the original and shuffled curves is wider than that between the original and surrogate ones. This suggests that the observed multifractality mainly stems from long-term correlations in the data, rather than from heavy-tailed distributions.

### The relationship between the temporal evolution of Δ***h*** and monthly FVC

**Figure 5:**
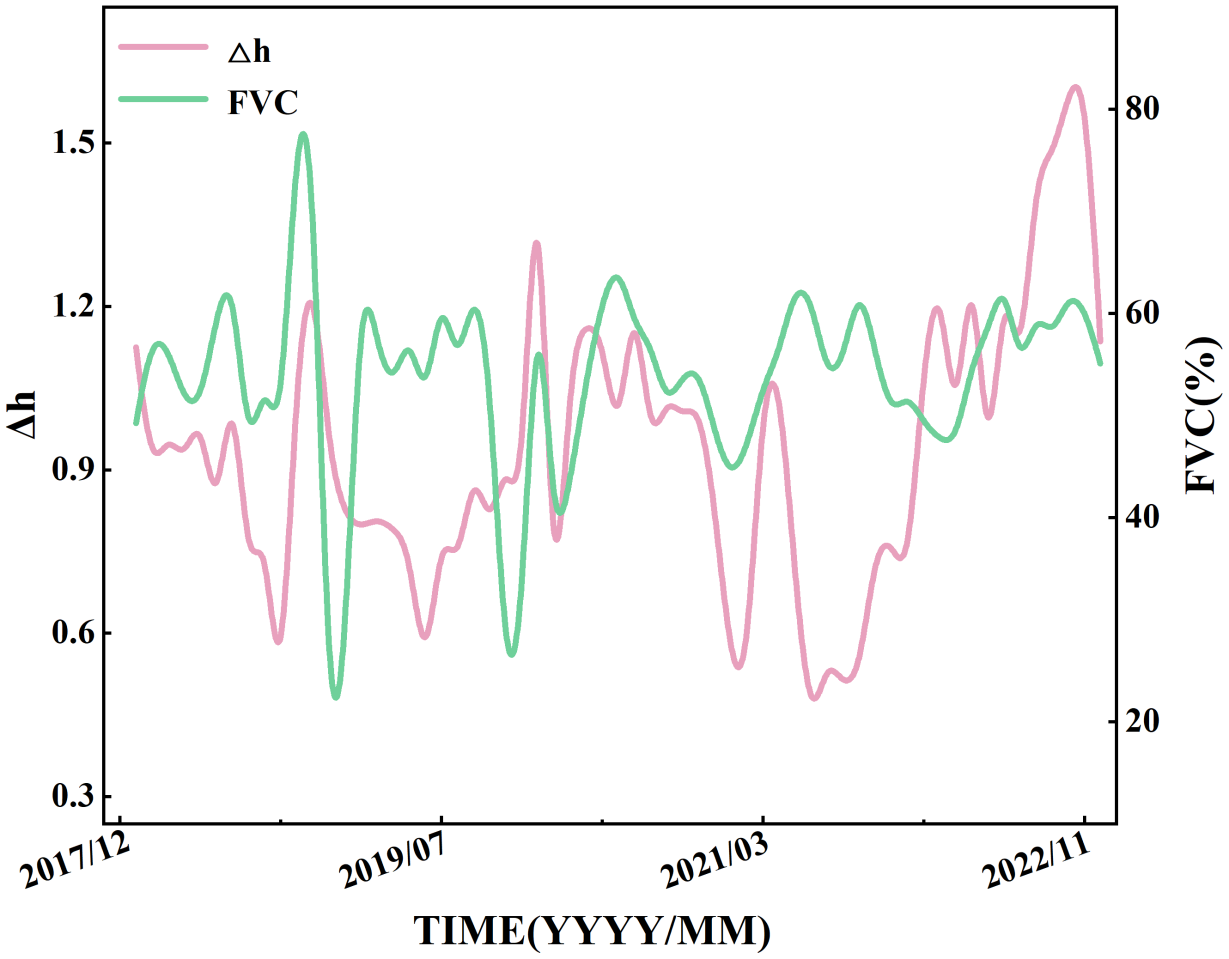
Temporal variations of the sliding-window water retention capacity (Δ*h*) and monthly fractional vegetation cover (FVC) in the Quxi River Basin, Chongqing, China, from 2017 to 2022.

Based on six years of precipitation and runoff data from 2017 to 2022, the MF-DCCA method was employed to quantify the multi-scale interactions between precipitation and runoff. Over long time scales, the interaction between precipitation and runoff is unlikely to remain constant; instead, it may evolve temporally. Calculating a single Δ*h* value for the entire period fails to capture the temporal heterogeneity of the interaction. Therefore, it is necessary to use a sliding window approach to analyze the temporal evolution of Δ*h* values between precipitation and runoff. The specific procedure is as follows: first, precipitation and runoff data from January 1, 2017, to December 31, 2017, were selected as the initial window, and the three highest-frequency intrinsic mode functions were extracted for subsequent analysis. Using the MF-DCCA method, the first Δ*h* value is calculated. Then, by advancing the time window one month at a time—while keeping its length fixed—subsequent Δ*h* values are obtained. Repeating this step resulted in a total of 61 values. Figure 5 shows the trend of Δ*h* over the entire study period, illustrated by the pink line.

FVC, a commonly used metric of vegetation activity, effectively reflects temporal variations in vegetation conditions. Exploring the relationship between Δ*h* and monthly FVC helps to reveal how vegetation influences the runoff response across multiple time scales. To this end, we analyzed their co-evolution over time, as shown in Fig. 5. Monthly FVC values were temporally averaged and aligned with Δ*h* to reduce short-term noise (*e.g.*, from cloud cover or rainfall), allowing a clearer view of vegetation’s longer-term impact.

As shown in Fig. 5, both Δ*h* and monthly FVC exhibit clear intra-annual periodic fluctuations.

**Figure 6:**
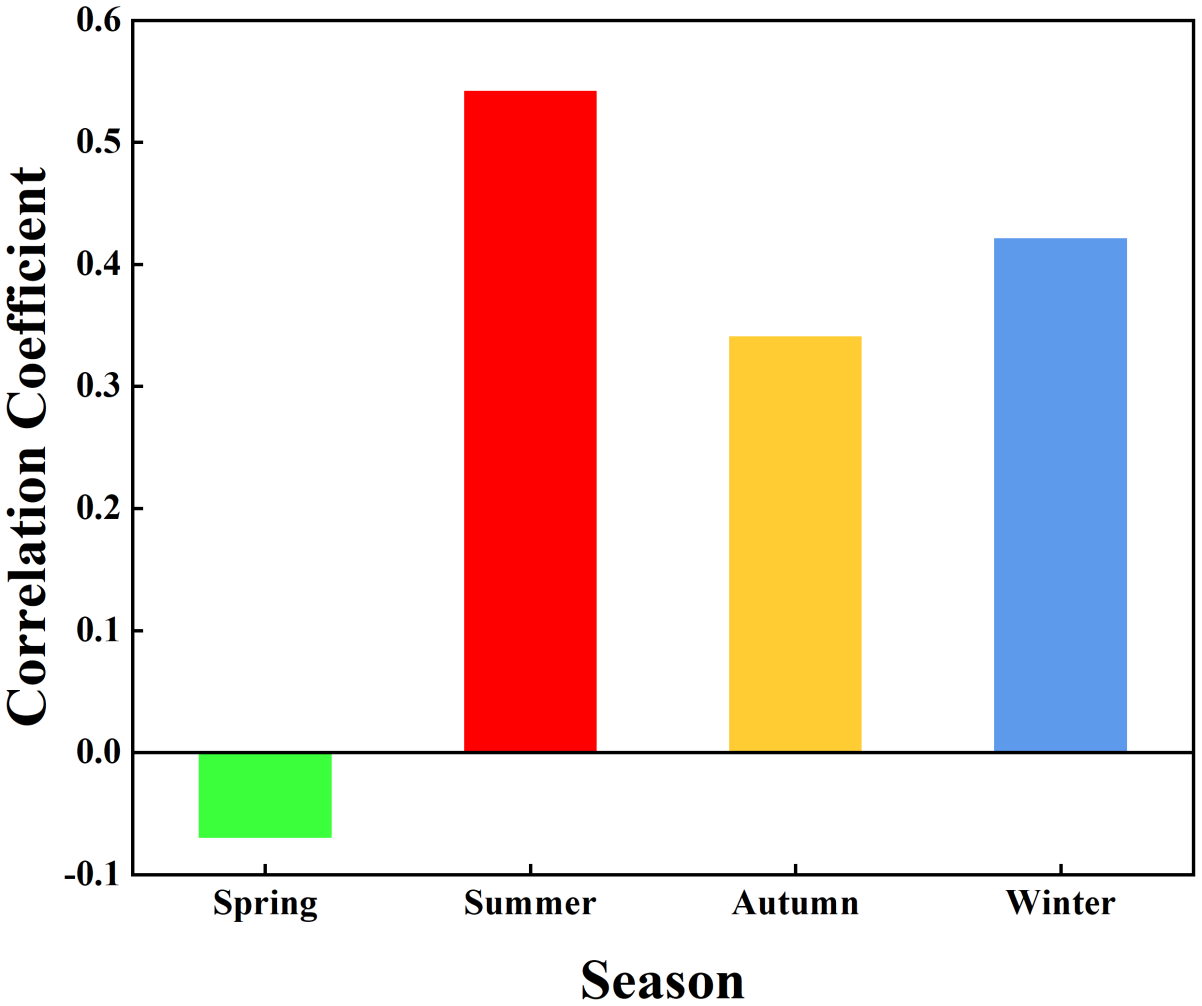
Pearson correlation coefficients between Δ*h* and FVC for the four seasons in the Quxi River Basin, Zhongxian County, Chongqing, China.

To further analyze the impact of vegetation dynamics on the hydrological system and quantitatively examine the relationship between Δ*h* and FVC across seasons, data from spring (March–May), summer (June–August), autumn (September–November), and winter (December–February of the following year) were extracted, and Pearson correlation analysis was performed. Fig. 6 presents the seasonal correlation coefficients between Δ*h* and FVC in the Quxi River Basin during the study period. The correlation coefficients for each season are as follows: spring (−0.07), summer (0.54), autumn (0.34), and winter (0.42). Results of the *t*-test indicate that the correlation coefficients differ significantly among the four seasons (*p* < 0.05).

**Figure 7:**
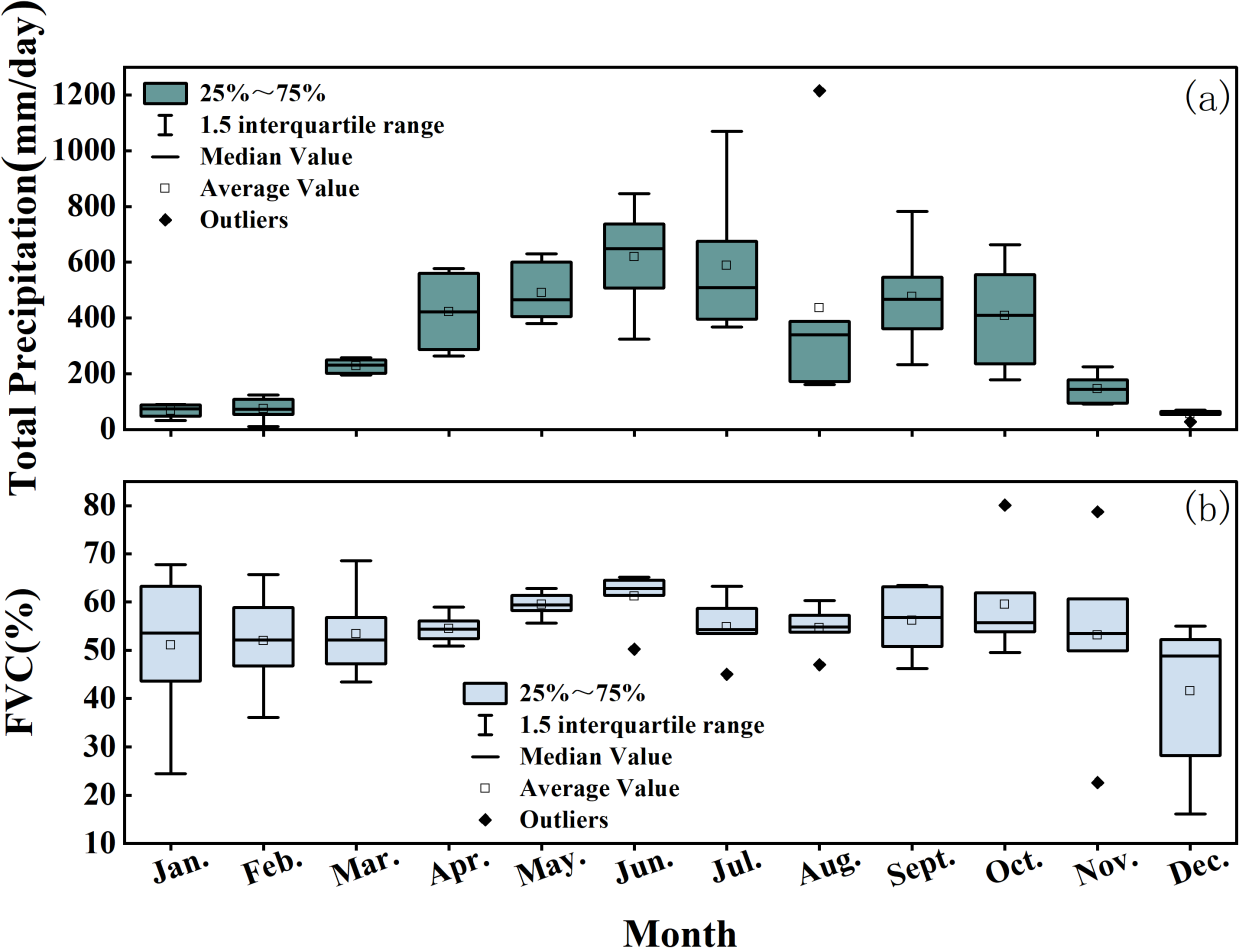
Monthly average statistical results of total precipitation and FVC in the Quxi River Basin, Zhongxian County, Chongqing, China.

In a typical process of natural succession, the monthly average FVC values generally exhibit seasonal fluctuations, with higher values in summer and lower values in winter. As shown in Fig. 7, the monthly average FVC values throughout the year show a relatively small variation and exhibit a stable seasonal trend.

## Discussion

### Short- and long-term variability revealed by EEMD

In the EEMD results, short-period components are more sensitive to local factors than long-period components in small watersheds. This may be related to the evapotranspiration regulation of local vegetation, groundwater infiltration, and its rejoining of surface runoff, as well as the low-frequency irrigation patterns for crops such as citrus (Atwood et al., 2023; García-Tejero et al., 2011; Xiao et al., 2024).

A study by Tian et al. (2020) in the southern valleys of the Tibetan Plateau showed that local evaporation triggers short-term hydrological processes, typically occurring on timescales of several days. In contrast, long-period components reflect the influence of large-scale factors, such as changes in moisture transport driven by the Asian summer monsoon at interannual scales and ecological responses to land use policies like reforestation over multi-year ecological responses (Xu et al., 2023). These factors exert a regulatory effect on local hydrological processes.

### Differences in persistence between runoff and precipitation revealed by DFA

In the DFA results, precipitation behaved as a near-random process, whereas runoff exhibited strong long-term persistence. These findings are consistent with those of Kantelhardt et al. (2006), who proposed that the long-term persistence of runoff is related to the soil water retention capacity. The lack of persistence in precipitation is primarily attributed to the stochastic nature of precipitation events and atmospheric dynamics. This conclusion provides a theoretical foundation for interpreting the long-term persistence of runoff in the present study.

A strong long-term persistence in runoff suggests that small watersheds tend to exhibit slower hydrological responses to extreme precipitation events than large watersheds, meaning that short-term rainfall is less likely to induce fast fluctuations in runoff. However, over long time scales, precipitation is gradually converted to runoff through the watershed regulation process, continuously replenishing the hydrologic system. The strong long-term persistence of runoff indicates a strong capacity for water conservation and effective regulation of water resources. On the contrary, a weak long-term persistence of runoff indicates a high sensitivity to changes in precipitation. In this case, runoff responds quickly and strongly to random precipitation events, reflecting the watershed’s poor water retention capacity. Based on a study of 605 streams and 696 annual runoff records older than 80 years, Markonis et al. (2018) found that the stronger the long-term persistence of runoff, the more stable the hydrologic response and the greater the potential water retention capacity. Conversely, the weaker the long-term persistence, the faster the hydrologic response to precipitation, and the weaker the water retention capacity.

The uncertainty in runoff dynamics is likely influenced by vegetation effects. Vegetation improves the soil’s ability to retain water more effectively. For instance, tall trees sink their roots deeply into the soil, facilitating water capture and absorption, which supports water storage and enhances overall ecosystem moisture retention (Yang et al., 2022). Herbaceous plants, on the other hand, have many fine roots concentrated near the soil surface, resulting in lower water retention capacity (Löbmann et al., 2020). Li et al. (2018) found that trees stored two to three times more water than herbaceous plants. These differences in water storage capabilities among plant types help explain why runoff persistence varies significantly across ecological zones and time scales. Due to the complexity of these processes, a single persistence index is insufficient to capture the full picture, and a multifractal approach provides more comprehensive information.

### Multiscale coupling and water retention capacity revealed by MF-DCCA

In the MF-DCCA results, the cross-correlation between precipitation and runoff exhibits a power-law scaling behavior. This indicates that, over certain time scales, the temporal evolution of their cross-correlation does not follow a classical Markov process but instead decays gradually in a power-law form. Therefore, the cross-correlation between precipitation and runoff exhibits multifractal scale invariance, reflecting a consistent relationship across temporal scales.

In the multifractal spectrum, a higher Δ*h* value indicates greater variability and heterogeneity in the cross-correlation between precipitation and runoff across different time scales, indicating increased complexity of the hydrological response in the watershed. This complexity arises from the coupled influences of multiple factors, such as soil types, topographical features, and human activities within the watershed. These factors collectively shape the spatial distribution and transformation pathways of precipitation, giving rise to diverse water retention mechanisms. These mechanisms improve water storage and regulation capacity in small watersheds across different spatial and temporal scales and reflect the nonlinear and multiscale characteristics of hydrological processes.

**Figure 8:**
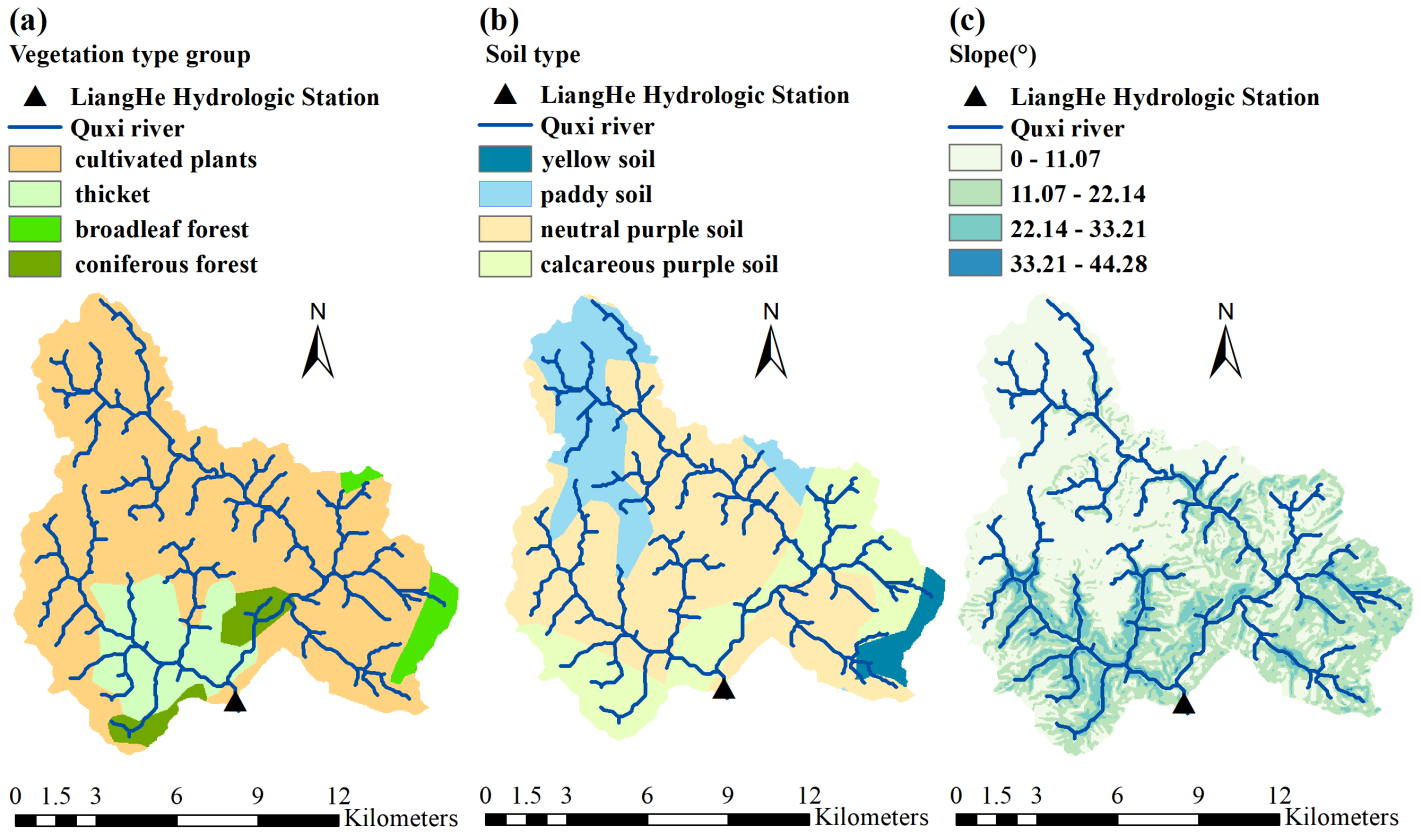
Spatial distribution of (A) vegetation types, (B) soil types, and (C) slope in the Quxi River Basin, Zhongxian County, Chongqing, China.

The complex hydrological response of the Quxi River Basin results from both natural and human influences. As shown in Fig. 8A, the basin is largely covered with cultivated vegetation (79.32%), with citrus trees being the most common. These trees have dense and highly absorptive root systems that enhance water retention, reduce surface evaporation, improve the soil’s ability to retain moisture, and promote rainfall infiltration (Kadyampakeni et al., 2014). As shown in Fig. 7B, the average monthly vegetation cover from 2017 to 2022 remained above 50%. Dense vegetation plays a key role in reducing surface runoff, encouraging water to soak into the ground, and aiding groundwater recharge.

As shown in Fig. 8B, the most common type of soil in the Quxi River watershed is neutral purple soil, accounting for about 54.87% of the total land area. Purple soils are often very permeable, especially on gentle slopes where rainwater is absorbed and retained (Wang et al., 2024). As shown in Fig. 8C, the basin has a generally gentle slope, which reduces surface runoff and favors rainfall infiltration, plant uptake, and underground storage. Gently sloping sites can reduce the impact of heavy rainfall, protect the structure of the soil, and help the soil hold moisture.

A stable and interactive soil-vegetation-topographic system has formed in the Quxi River Basin. This system is formed by extensive citrus cultivation, dense surface vegetation, well-drained purple soil, and gentle terrain. These characteristics jointly help the basin retain moisture at different temporal and spatial scales, promote infiltration, and slow down runoff. Therefore, the hydrological response becomes more complex and varies with scale, while the overall water retention capacity of the small watersheds increases. In this study, the Δ*h* parameter in the multifractal spectrum was used to describe this complexity. The larger the Δ*h*, the higher the water storage efficiency and the slower the release rate, and correspondingly, the stronger the retention. Therefore, Δ*h* can be used as a key indicator for evaluating the water-holding capacity of a basin.

The Δ*h* value of precipitation and runoff in the Quxi River Basin is 1.10, indicating a strong multifractal intensity of cross-correlation between precipitation and runoff. This suggests that the transformation process between precipitation and runoff is relatively complex, and the basin has a strong water retention capacity.

Generally, there are two main sources of multifractal characteristics in time series. One is the presence of sharp spikes and heavy-tailed probability distributions of the time series, and the other is the long-term persistence of fluctuations at different time scales (Shi, Liu & Huang, 2015). Phase randomization and random shuffling are commonly used to determine the contributions of these sources to the multifractal properties. Phase randomization preserves the linear properties of the original time series while disrupting its non-Gaussian features, thereby identifying the contribution of sharp spikes and heavy tails to the multifractal characteristics. In contrast, random shuffling retains the original data values but eliminates the inherent temporal correlations, allowing an assessment of the contribution of long-term persistence to the multifractal properties (Li et al., 2024b).

### Influence of vegetation cover on water retention capacity (Δ*h*)

The first three IMFs extracted through the EEMD method exhibited high variance and short periodicity, indicating that short-term processes dominated the precipitation–runoff dynamics in the Quxi River Basin. Among these processes, vegetation dynamics play a key role in influencing precipitation–runoff interactions through evapotranspiration, interception, and infiltration (Ajami et al., 2017). Therefore, the first three IMFs were selected for the sliding-window analysis of the temporal evolution of Δ*h* between precipitation and runoff.

The annual cyclic fluctuations observed in Δ*h* and monthly FVC indicate a strong relationship between the seasonal dynamics of vegetation cover and the multi-scale interactions of precipitation and runoff. The observed rise–fall–rise pattern in monthly FVC from October to December of 2018 and 2019 may be closely related to citrus ripening, anthropogenic interventions such as pruning, and subsequent regrowth of vegetation in the Quxi River Basin.

In summer, Δ*h* and FVC exhibited the strongest positive correlation. This suggests that vegetation can effectively enhance hydrological response during the summer. Summer, characterized by the highest precipitation and vigorous vegetation growth, exhibits the strongest hydrological response, as vegetation influences water dynamics through multiple pathways. First, the main cultivated plant in the Quxi River Basin is citrus trees. During the summer, abundant precipitation combined with dense foliage and a large leaf area in citrus trees promotes substantial rainfall interception, effectively slowing the rate at which precipitation reaches the ground and enhancing soil infiltration. The root system of citrus trees has a high water demand, and the ample summer rainfall promotes rapid root growth, loosening the soil and increasing soil porosity. Some of the moisture is absorbed by the plants, while the rest replenishes groundwater, effectively conserving water resources and influencing hydrological response. Additionally, high summer temperatures and strong transpiration lead to the release of large amounts of moisture into the atmosphere, significantly increasing near-surface air humidity. This potentially facilitates local convective precipitation in the small catchment. Under the influence of leaves and roots, vegetation slows the transformation of precipitation into surface runoff, impacting the hydrological response and enhancing the water retention capacity of the small catchment. The summer results are consistent with previous studies in the headwater regions of the Yangtze and Yellow Rivers, where summer vegetation is jointly regulated by high leaf area index, large vegetation coverage, and abundant precipitation. In these areas, precipitation interception reaches 8.80–10.40 mm, demonstrating strong water retention capacity, which is significantly positively correlated with vegetation coverage (Li et al., 2025).

In autumn, Δ*h* and FVC exhibited a weaker positive correlation. As temperatures decline and precipitation decreases, the growth of citrus trees slows down, root activity diminishes, and the water conservation capacity weakens, thereby affecting hydrological response. Additionally, as citrus fruit gradually ripens in autumn, more nutrients and moisture are directed toward the fruit rather than leaf growth. As the leaves undergo natural senescence, the resulting reduction in leaf area diminishes the tree’s ability to intercept precipitation, further weakening the hydrological response. However, the pruned leaves accumulate on the ground, which slows down the rate at which precipitation reaches the surface, increasing moisture infiltration and reducing surface runoff.

In winter, Δ*h* and FVC exhibit a stronger positive correlation. Although precipitation in the Quxi River Basin is relatively low in winter, citrus trees, which are evergreen, still maintain a substantial leaf area, which provides some precipitation interception capacity. Some of the precipitation is intercepted and infiltrates into the soil for storage, influencing the hydrological response. On the other hand, the Quxi River Basin experiences lower temperatures and higher altitudes in winter, with occasional snowfall. During snow events, the tree canopy can intercept snow and reduce incoming solar radiation, resulting in lower temperatures beneath the canopy. This delays snowmelt, and the process of converting meltwater into surface runoff is significantly slowed, thereby enhancing the water retention effect and modifying the hydrological response of the small catchment.

In spring, Δ*h* and FVC exhibit a very weak negative correlation. This is because, with rising temperatures in spring, snow melts rapidly, and vegetation grows slowly, while a large amount of meltwater is released in a short period. Vegetation can only intercept a portion of the meltwater, leading to a rapid generation of surface runoff and affecting the hydrological response of the small catchment.

As shown in Fig. 7A, the average total precipitation in spring is 1,141 mm, and in summer it is 1,642 mm. Although precipitation in both seasons exceeds 1,100 mm and is relatively high, there is a significant seasonal difference in the influence of vegetation dynamics on the water retention capacity of the small watershed. This can be explained by several factors. This is primarily because the vegetation in the Quxi River Basin is dominated by cultivated plants, which account for 79.32% of the total watershed area, with citrus orchards being the main type. In spring, citrus leaves are just beginning to develop, and the canopy is sparse, resulting in a leaf area index much lower than in summer (Wang & Guo, 2024). Consequently, rainfall interception by vegetation is limited, and its regulatory capacity on hydrological processes is weak. Second, water in spring is primarily used for vegetative growth and flower bud development, whereas in summer, a larger portion is allocated to fruit growth (Agustí et al., 2022). Studies indicate that the daily water requirement of citrus in summer is 74%–89% higher than in spring, with increased water uptake by the roots, enhancing the regulatory effect of vegetation (Al-Qthanin et al., 2024). Finally, citrus trees are still in the early stage of growth in spring, making them less capable of mitigating rapid hydrological responses caused by snowmelt and precipitation associated with rising temperatures. As a result, the influence of vegetation on watershed water retention in spring is minimal and may even be negative.

In this study, the FVC values in winter are relatively low, which may be related to the winter pruning of branches and leaves. This also reflects the land use characteristics of the area, which are primarily dominated by fruit tree cultivation.

The assessment of the water retention ecological service function has received widespread attention in previous studies. Some studies have applied improved hydric function indices to evaluate water retention capacity; however, these methods do not account for the seasonality of precipitation, extreme precipitation events, or soil moisture saturation, and therefore have certain limitations (Šatalová & Kenderessy, 2017). Other studies have assessed water retention functions based on the comprehensive water-storage capacity method; nevertheless, this approach requires a clear understanding of forest types and hydrological mechanisms (Shi et al., 2021). In addition, estimating watershed water retention capacity based on the area of low-lying regions and storage depth can be affected by inherent errors in digital elevation models (DEMs) (Kessler & Gupta, 2015).

The method proposed in this study is not subject to the aforementioned limitations; it can not only quantify the water retention capacity of small watersheds but also comprehensively reflect the complex effects of vegetation dynamics on hydrological responses.

Analysis of Δ*h* and FVC over time suggests that Δ*h* not only measures the water retention capacity of small catchments but also captures the complex effects of vegetation dynamics on hydrological responses. Incorporating Δ*h* into the quantitative assessment of ecological water retention functions allows for more accurate prediction and assessment of hydrological behavior and ecosystem services. This method lays a strong foundation for sustainably managing small watershed ecosystems and supports efforts to protect and restore their ecological functions.

## Conclusions

In this study, we applied EEMD, DFA, and MF-DCCA to analyze runoff observations, remotely sensed precipitation, and vegetation cover in the Quxi River watershed, Zhongxian County, Chongqing, China, from 2017 to 2022. The findings suggest that changes in vegetation cover within sub-basins influence hydrological responses.

The EEMD analysis for the Quxi River watershed revealed that the top three high-frequency modes of precipitation and runoff exhibit an average cycle length of about two weeks. These components collectively explained a significant share of the total data variance. This means that the combined high-frequency modes can effectively represent the main patterns of short-term fluctuations in precipitation and runoff. According to DFA analysis, precipitation in the Quxi River Basin behaves as a purely random process without long-term persistence, whereas runoff exhibits long-term persistence. Thus, there is a significant difference between the precipitation and runoff sequences.

The MF-DCCA analysis revealed that the coupling relationship between precipitation and runoff exhibits multifractal characteristics across multiple time scales. This indicates that the multifractal features between precipitation and runoff can quantitatively describe the cross-correlation strength, which is closely related to the hydrological response of the small catchment. Based on this, the multifractal parameter Δ*h* was used to describe the hydrological response of the small catchment. The time evolution of Δ*h* was shown to support the feasibility of using Δ*h* to characterize the hydrological response of the small catchment. The correlation coefficients between Δ*h* and FVC in the spring, summer, autumn, and winter seasons were −0.07, 0.54, 0.34, and 0.42, respectively. The results confirmed that vegetation can effectively moderate the hydrological response of the small catchment, especially in summer. Further analysis showed that the seasonal differences in this effect were mainly due to the differences in vegetation dynamics across seasons.

This study presents a novel approach that integrates remote sensing precipitation imagery, ground-based runoff observations, and vegetation coverage data. By combining the multifractal analysis method with vegetation dynamics, the variation in water retention capacity of small watersheds under changing vegetation conditions is quantitatively assessed using the Δ*h* value. This method not only quantifies the regulatory effects of vegetation dynamics on watershed hydrological processes but also serves as an important indicator for evaluating the ecological service function of small watersheds. In the context of increasingly frequent extreme weather events and the pursuit of sustainable development, a safe, rapid, and accurate identification and quantitative evaluation of watershed water retention capacity can support vegetation restoration and ecological function improvement, thereby enhancing species diversity within small watersheds. The assessment of water retention capacity also provides scientific guidance for forest management, such as implementing timely thinning or understory vegetation clearing, to prevent ecological vulnerability and sensitivity caused by excessively strong or weak water retention capacity, and to maintain the stability and sustainability of watershed ecosystems. Furthermore, this method exhibits strong adaptability and can be extended to various small watersheds influenced by the construction of large hydraulic engineering projects. It can assist decision-makers in promptly identifying the degradation trends of watershed ecological functions and implementing targeted ecological restoration and remediation measures, thereby promoting the coordinated advancement of watershed ecological protection and sustainable development.

##  Supplemental Information

10.7717/peerj.20496/supp-1 Supplemental Information 1 Original precipitation and runoff data

10.7717/peerj.20496/supp-2 Supplemental Information 2 Figure 2 data

10.7717/peerj.20496/supp-3 Supplemental Information 3 Figure 3 data

10.7717/peerj.20496/supp-4 Supplemental Information 4 Figure 4 data

10.7717/peerj.20496/supp-5 Supplemental Information 5 Figure 6 data

10.7717/peerj.20496/supp-6 Supplemental Information 6 Figure 7 data

10.7717/peerj.20496/supp-7 Supplemental Information 7 Figure 8 data

## Additional Information and Declarations

### Data Availability

The following information was supplied regarding data availability: The raw precipitation and runoff data are available in the Supplemental Files.
